# Strain behavior of native and reconstructed medial patellofemoral ligaments during dynamic knee flexion - a cadaveric study

**DOI:** 10.1186/s40634-019-0195-3

**Published:** 2019-07-03

**Authors:** Mohamed Buhary Kizher Shajahan, Chaw Tat Alex Choh, Khye Soon Andy Yew, Hiok Yang Chan, Tet Sen Howe, Tjiauw Tjoen Denny Lie, Suang Bee Joyce Koh, Chee Cheng Paul Chang

**Affiliations:** Department of Orthopaedic Surgery, Singapore General Hospital, 20 College Road, Singapore, 169856 Singapore

**Keywords:** MPFL, Reconstruction

## Abstract

**Background:**

Surgical reconstruction of the Medial Patello-Femoral Ligament (MPFL) has been recognized as an effective treatment for patients with instability despite conservative treatment. The purpose of this cadaveric study is to compare the strain patterns within the native and reconstructed single and double-bundle MPFL. This will help ascertain if the native biomechanics are restored with the reconstructions.

**Methods:**

Twelve cadaveric knees were dissected and the native MPFL of each specimen was identified. The knees were subjected to dynamic flexion using a customized jig. Continuous strain measurements were taken for each knee from 0 to 120 degrees flexion and then back to full extension using differential variable reluctance transducers (DVRTs). The MPFL was then cut. Six single bundle and six double bundle MPFL reconstructions were performed using hamstring tendon grafts. The DVRTs were reattached to the grafts and strain measurements were retaken. Statistical analysis was performed using a paired t-test.

**Results:**

Strain patterns of the native and reconstructed MPFL showed an increase in strain from 0 to 120 degrees of flexion except for the inferior bundle of the double bundle reconstruction. The strain patterns in the intact specimens were higher than the reconstructed MPFL through different degrees of knee flexion. In the double-bundle group, the superior graft had statistically significantly lower strains compared to the native MPFL with *p*-value <.05 at all flexion angles. The reconstructed inferior band showed loss of tension as the knee flexed. Higher strain with statistical significance (*p*-value <.05) was found in the single-bundle compared to the superior band of the double-bundle reconstruction at flexion angles less than 90 degrees.

**Conclusion:**

The strain variation at progressive angles of knee flexion is dissimilar between the native and reconstructed MPFL. The reconstructed MPFL exhibited non-physiological biomechanics with the inferior band losing tension. Although the single-bundle reconstruction shows a better strain profile compared to double-bundle reconstruction, the grafts are significantly stiffer than the native MPFL.

## Background

Patellar instability is a disabling condition that affects a large number of patients, particularly females 10 to 17 years old. Patients who present with chronic patella dislocation are more likely to be female, are older, and have a greater risk of subsequent patellar instability than first-time dislocators (Conlan et al. 1993; Fithian et al. 2004). It can lead to long-term problems such as cartilage injury and functional limitation (Fithian et al. 2004; Cofield and Bryan 1977; Hawkins et al. 1986). Patients with predisposing factors such as patellofemoral malalignment, abnormal patellar configuration, and history of prior symptoms of instability were more prone to recurrent dislocations and may benefit from operative intervention. The MPFL acts as the primary stabilizer of the patella in early flexion angles and is ruptured in 95–100% of chronic patellar instability cases (Conlan et al. 1993; Amis et al. 2003; Placella et al. 2015).

During knee flexion and extension, the patella stability is maintained by soft tissue and bony structures. In full extension of the knee, the patella sits anterior to the distal femur, with only the distal pole of the patella in contact with the superior trochlea groove. At this time, the medial patellofemoral ligament (MPFL) is taut and prevents lateral subluxation of the patella (Philippot et al. 2009). Between 15 and 30 degrees of knee flexion, the MPFL tightens and the patella begins to engage the trochlea. The lateral femoral condyle and medial soft tissue stabilizers prevent lateral subluxation of the patella. (Alemparte et al. 2007; Tuxoe et al. 2002).

Surgical reconstruction of MPFL has been recognized as an effective treatment for patients with functional or symptomatic instability despite conservative treatment (Enderlein et al. 2014; Howells et al. 2012; Mackay et al. 2014; Schneider et al. 2016; Smith et al. 2007; Stupay et al. 2015; Tompkins and Arendt 2015). A systemic review by Mackay et al. concluded that the meta-analysis of 17 case series shows that MPFL reconstruction alone has resulted in improvement in Kujala scores, a low dislocation rate in recurrent patellar dislocation (Mackay et al. 2014). It has been shown that the anatomical placement of the reconstructed MPFL leads to better isometry and patellofemoral kinematics (Philippot et al. 2009; Schneider et al. 2016; Stephen et al. 2014; Stephen et al. 2016; Stephen et al. 2012). Stephen et al. demonstrated that anatomical placement of grafts resulted in the restoration of medial joint line contact pressures and patellar tracking using three different grafts (gracilis, quadriceps and tensor fascia latae) for the reconstruction of the MPFL. They also showed that femoral tunnel placement, either too distal or proximal resulted in an increase in medial joint line pressures and thus altering the kinematics of the patella (Stephen et al. 2012). Although several studies have analysed the change in length, load to failure and tensile strength of the native MPFL (Amis et al. 2003; Stephen et al. 2014; Kim et al. 2014; Mountney et al. 2005), there is just one study that looks at the strain behavior of native MPFL and the reconstructed MPFL.

McCulloh et al. compared the strain within the MPFL, medial retinaculum and reconstructed MPFL using a visible-light stereophotogrammetry system (McCulloch et al. 2014). They measured the strain at various angles of knee flexion and concluded that native strains were not reproduced after reconstruction and was found to be significantly lower. Their study did not compare the strain within the two bundles of the reconstructed MPFL nor were the knees tested in dynamic flexion but at various static flexion angles.

Understanding the strain patterns in dynamic knee flexion will help identify the optimal reconstruction technique to replicate the native strain behavior and restore patellofemoral kinematics. Double and single bundle reconstructions are recognized as effective treatments for reducing recurrent patella dislocations (Kang et al. 2010; Ellera Gomes 1992).

### Aim

This study aims to evaluate the strain behaviour of the native MPFL and compare the strain behaviour with the single and double-bundle reconstructed MPFL in dynamic knee flexion. The null hypothesis is that current MPFL reconstruction techniques do not replicate native strain patterns.

## Methods

### Specimen preparation

Twelve fresh-frozen, adult cadaveric knees (average age 55, range 46 to 67 years) with no previous surgeries were obtained from a tissue donor bank. The knees were physically examined and those with significant osteoarthritis and ligamentous laxity were excluded from the study. Arthrotomies were performed after the experiment to confirm no trochlea dysplasia and osteoarthritis. Specimens consisted of the mid-femur and mid-tibia with their respective muscle attachments. The specimens were stored at − 20 °C before use, thawed at 4 °C overnight and then at room temperature for six hours on the day of the experiment. A midline incision was made over the knee joint with careful dissection of the skin and subcutaneous fat. The medial and lateral retinaculum was preserved. The MPFL was identified and the femoral attachment was marked.

### Testing protocol

**Fig. 1:**
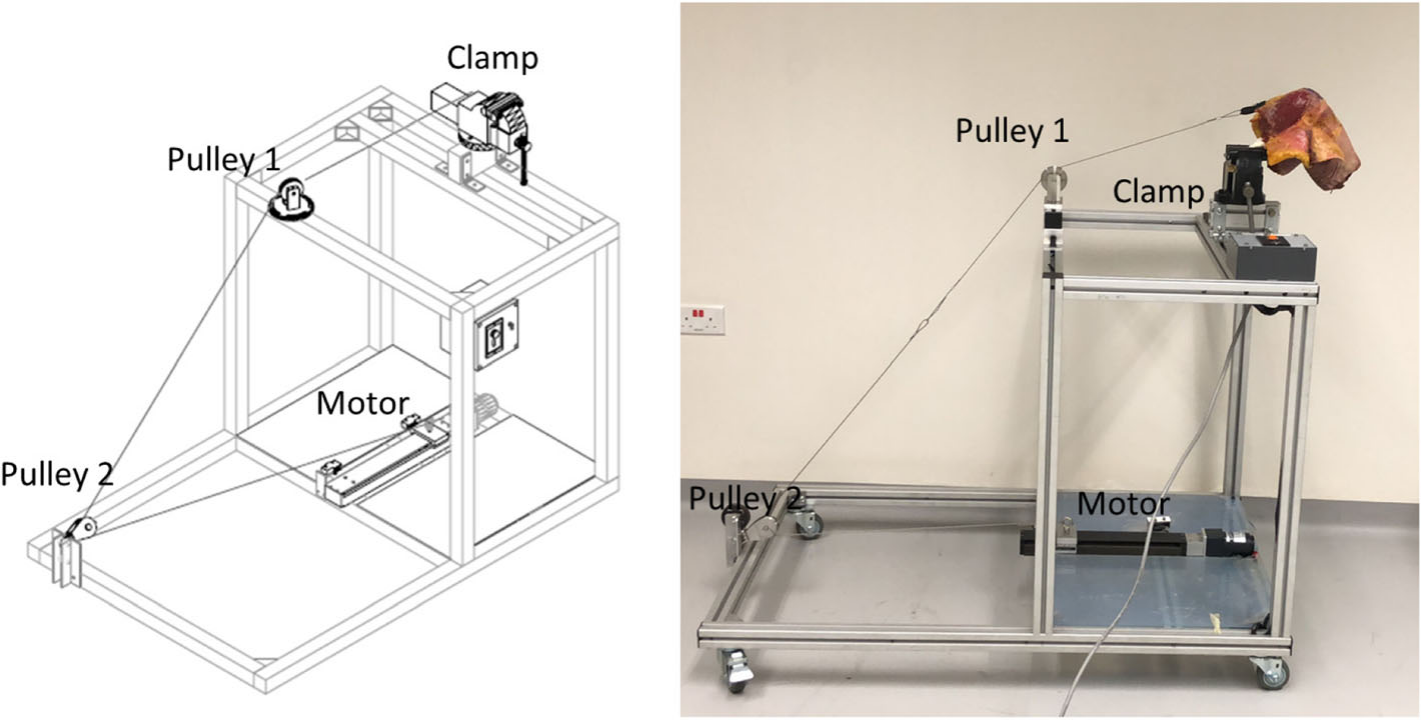
Customized Jig showing the clamp for attachment of femur, pulleys and the motor

Each knee was mounted onto a customized jig. The jig (Fig. 1) consisted of a clamp to secure the distal femur, a pulley to pass the cables, and a motor to pull on the cables. The femur was securely clamped at an angle with respect to a freely hanging tibia in order to position the knee at 120 degrees flexion. All native knees were able to achieve 120 degrees of flexion.

**Fig. 2:**
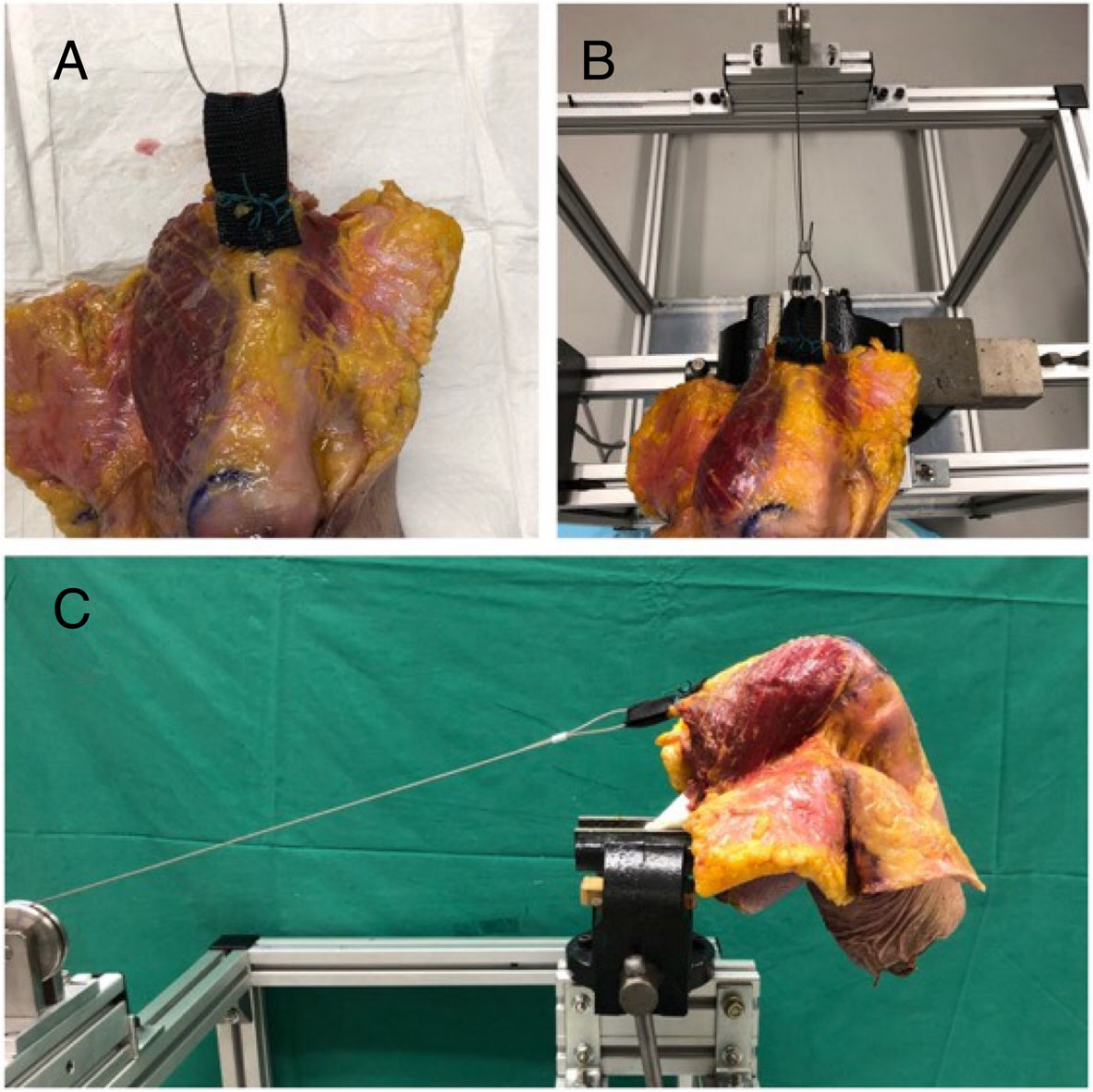
**A** – Nylon band attached the quadriceps tendon **B** – Cable attached to nylon band **C**- Femur mounted onto jig with cable running through pulley system

The quadriceps muscle was dissected and a nylon strap was sutured onto the tendinous portion of the quadriceps muscle creating a loop (Fig. 2a). A stainless-steel cable of 3 mm diameter was passed through the loop. The wires were passed through a pulley system to a linear motor which moved at a constant velocity of 3.33 mm/s.

The tibia was then elevated to full extension by the quadriceps pull stemming from the motion of the linear motor. To flex the knee, the direction of the motor motion was reversed and the tension on the cable was released.

An electromagnetic (EM) sensor was placed longitudinally on the tibia, 20 mm distal to the tibial tubercle, and secured with a single suture to track the flexion angle dynamically.

**Fig. 3:**
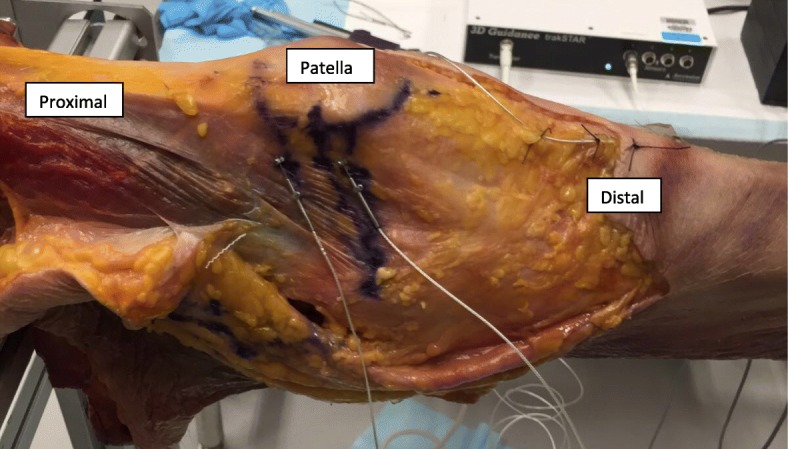
DVRT attached to the native MPFL of left knee, Electromagnetic sensor attached distal to tibial tubercle

The sail-like shape of the intact MPFL was outlined. Then, a differential variable reluctance transducer (DVRTs) (LORD, MicroStrain® Sensing Systems, USA) was placed on both the superior and inferior aspect of the intact MPFL (Fig. 3), along the superior and inferior margins of the intact MPFL respectively. The anatomic femoral attachment (midpoint of the medial epicondyle and adductor tubercle) was also marked.

Throughout the study, continuous strain measurements (3 readings per second) were taken for each knee from 0 to 120 degrees flexion and then back to full extension. For each knee, strain measurements were taken three times as the knee extended and three times as the knee was flexed. The mean of these readings was used in our results. A mouse utility software (MurGee Softwares Pte Ltd., India) with a lag of 1 millisecond was used to ensure a near simultaneous start of data recording between the flexion angle and the strain measurements.

### Surgical reconstruction

Six of the twelve knees underwent an anatomical double-bundle MPFL reconstruction. Surgeries were performed by a surgical trainee under the supervision of a senior orthopaedic surgery consultant. The semitendinosus tendon was harvested from each of the knees. The tendon was debrided of muscle and cut into two grafts of equal length. The ends of each graft were whip-stitched 10 mm using monofilament sutures (Ethilon2/0; Ethicon Co). The intact MPFL was cut along the patella attachment taking note of the superior and inferior borders of the MPFL. An incision was made of the proximal two thirds of the medial aspect of the patella. A rongeur was used to create a 20 mm long, 5 mm wide and 3 mm deep trough on the medial border of the patella. The drill guide for the Arthrex 5.5 mm Bio-Tenodesis™ Screw was placed at the superior border of the MPFL (approximately 1 o’clock position on right knee, 11 o’clock position left knee) within the medial bone trough. A guide pin was over-drilled to a depth of 18 mm and a second drill guide was placed at the inferior border of the MPFL (approximately 3 o’clock position on right knee, 9 o’clock position left knee). Once drilled, both guide wires were removed. The tails of one graft end was passed through the eyelet of the Arthrex 5.5 mm Bio-Tenodesis™ Screw. The graft/anchor was pushed into the proximal drill hole until the eyelet is fully seated. The suture limbs were attached onto the driver to maintain tension. The screw was then tightened into the patella tunnel. After the driver was removed, the sutures were tied to reinforce the fixation. This was repeated for the second graft. The anatomic femoral attachment of the MPFL was identified by dissecting down the medial aspect of the knee as described by La Prade (LaPrade et al. 2007). The adductor tubercle and the medial epicondyle were identified and marked. A guide-wire was passed slightly posterior to the mid points of these two landmarks and then femoral tunnel was drilled (Nomura et al. 2005).

The sutured ends of the two grafts were passed through the femoral tunnel and pulled through the lateral side. Care was taken to ensure that the grafts were not twisted and both of the graft’s ends were passed through the tunnel. The knee was mounted on the jig to keep the knee flexed at 30 degrees. While keeping the lateral patellar facet flush against the lateral femoral condyle an Arthrex Bio-Interference Screw 7 mm was inserted into the femoral tunnel and the grafts tightened (Howells et al. 2012; Stephen et al. 2014; Stephen et al. 2016; Stephen et al. 2012; Kim et al. 2014; Russo et al. 2016). The knee was ranged to assess patella tracking and to ensure the grafts were not over-tensioned.

**Fig. 4:**
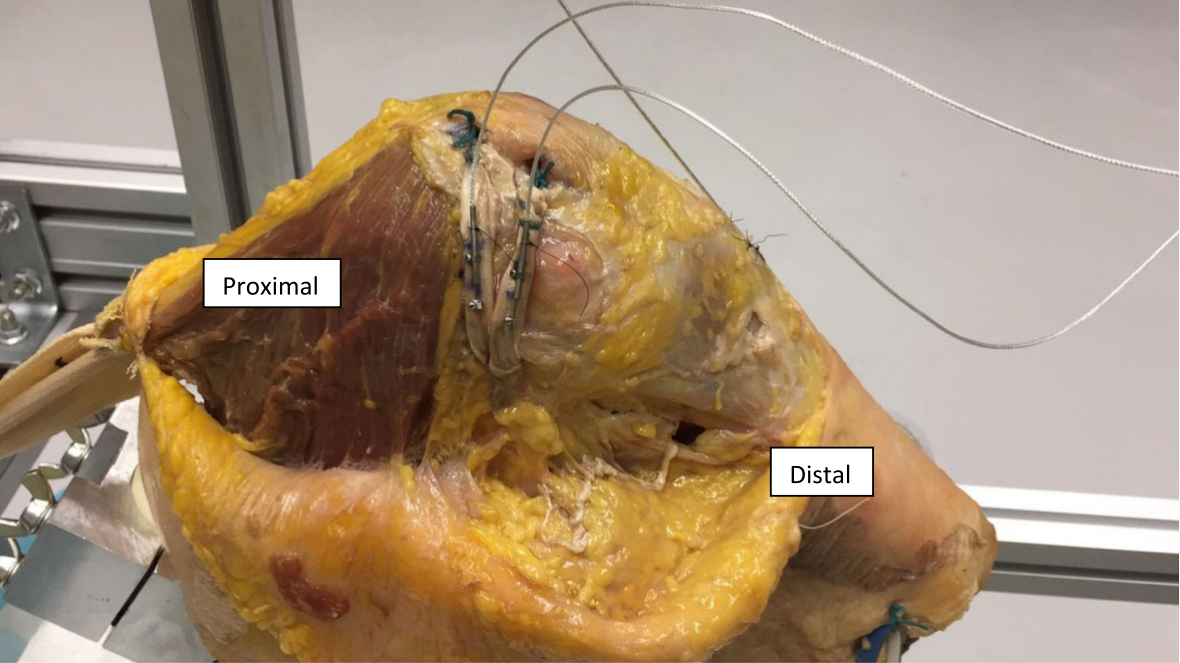
DVRT attached to double bundle reconstructed MPFL of left knee mounted onto the jig

The DVRT was reattached to the superior and inferior bundles of the reconstructed MPFL (Fig. 4). Similar to the intact MPFL testing, continuous strain measurements were taken for each knee from 0 to 120 degrees flexion and then back to full extension.

The remaining six knees underwent single-bundle MPFL reconstruction using the semitendinosus tendon graft. The Bio-tenodesis screw was placed at the midpoint of the native MPFL at the patellar insertion. The femoral tunnel placement and tensioning was done similar to the double-bundle group. A DVRT was attached to the single bundle and continuous strain measurements were taken for each knee from 0 to maximum flexion and then back to full extension.

For each knee, strain measurements were taken 3 times as the knee extended and 3 times as the knee was flexed. The mean of these readings was used in our results.

### Statistical analysis

Statistical analysis of the experimental data was carried out by comparing the mean strain values recorded from the intact and the reconstructed specimens in R (Version 3.22) using a Paired t-test. All comparisons were two-tailed and performed as a function of the anatomical location (superior, inferior) and flexion angle of the knee. *P*-values less than .05 were considered as statistically significant. Post-hoc power analysis was performed using G*Power 3.1.9.2.

## Results

### Intact MPFL superior VS inferior MPFL aspect

**Fig. 5:**
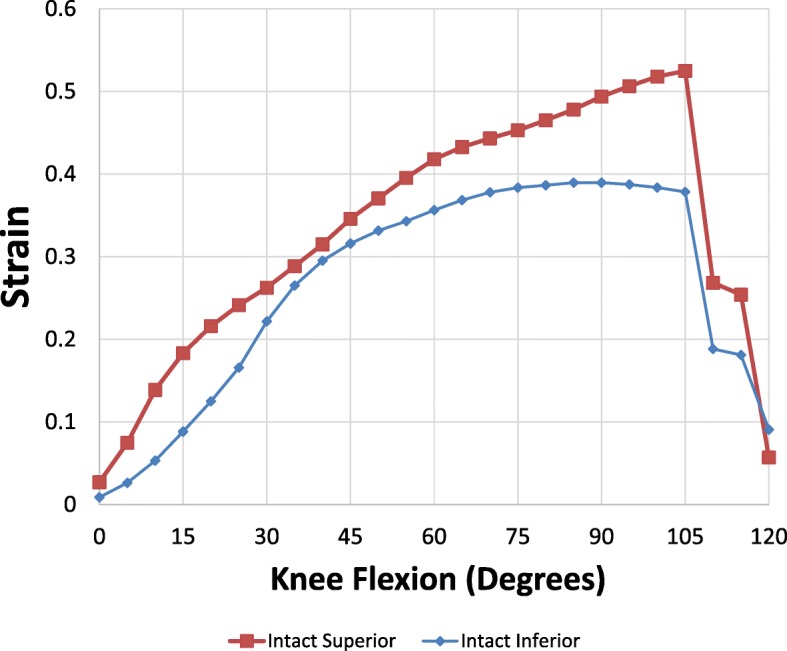
Mean strain pattern of intact (native) MPFL as knee flexed from 0 to 120 degrees. Red – superior intact, Blue – inferior intact

The mean strain pattern of the native MPFL (*n* = 12) when the knee flexed from 0 to 120 degrees is shown in Fig. 5. It was observed that the MPFL strain in both the superior and inferior aspects increased as the knee was flexed and peaked at 105 degrees of flexion. The most significant increase in strain was around 25–30 degrees of flexion. The strain over the superior aspect of the intact MPFL was more than the inferior aspect of the intact MPFL in all degrees of flexion. No statistical significance was observed between the superior and inferior aspects of the native MPFL throughout flexion.

### Intact MPFL VS double bundle reconstructed MPFL

**Fig. 6:**
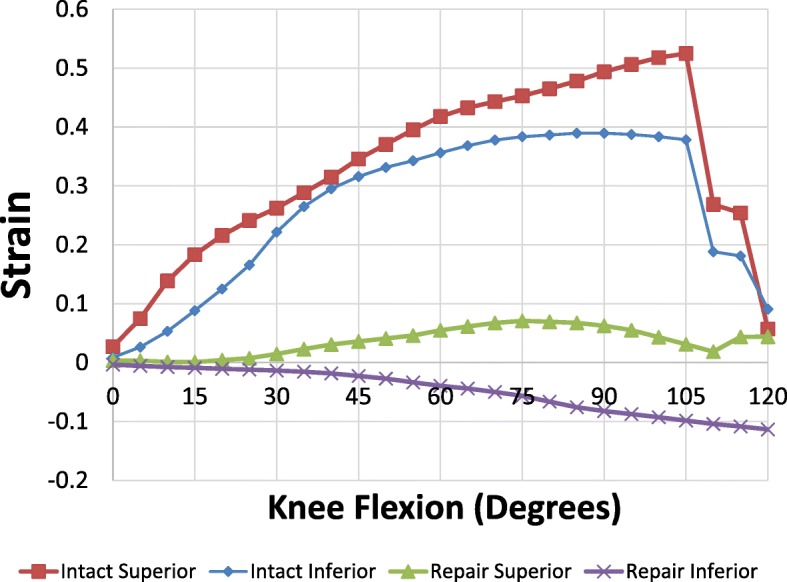
Strain pattern of intact and reconstructed MPFL as knee flexed from 0 to 120 degrees. Red – superior intact, Blue – inferior intact, Green – superior reconstructed, Purple – inferior reconstructed

Figure 6 shows the mean strain profile comparing the native MPFL (*n* = 12) and double-bundle reconstructed MPFL specimens (*n* = 6). In the double-bundle MPFL group, the superior band showed an increase in strain and then a decrease after 90 degrees of flexion. The reconstructed inferior band showed loss of tension exhibiting negative strain as the knee was flexed. The strain measurements in the intact specimens were significantly higher than the reconstructed MPFL at different degrees of knee flexion with statistical significance as reported in Table 1. For reconstructed MPFL specimens, statistically significant differences between the superior and inferior band at knee flexion 45 degrees (*P* = .016) and 60 degrees (*P* = .019) were observed.

**Table 1:** Superior and inferior MPFL strain for intact and reconstructed double-bundle knee specimens (*n* = 6)

	Superior			Inferior		
Flexion (Degrees)	Intact	Repair	*P*-value	Intact	Repair	*P*-value
30	0.262 ± 0.168	0.015 ± 0.031	.026	0.222 ± 0.127	−0.013 ± 0.011	.017
45	0.346 ± 0.155	0.036 ± 0.02	.012	0.316 ± 0.181	−0.023 ± 0.025	.017
60	0.418 ± 0.143	0.055 ± 0.018	.005	0.356 ± 0.205	− 0.039 ± 0.058	.02
90	0.494 ± 0.097	0.063 ± 0.062	.002	0.39 ± 0.2	−0.082 ± 0.15	.023
120	0.503 ± 0.145	0.018 ± 0.154	.013	0.359 ± 0.193	−0.114 ± 0.201	.027

For the six knees, the mean statistical power for the superior band and inferior band is 0.86 and 0.94 respectively. Statistical power above 0.8 is considered acceptable (Erdfelder et al. 1996).

**Fig. 7:**
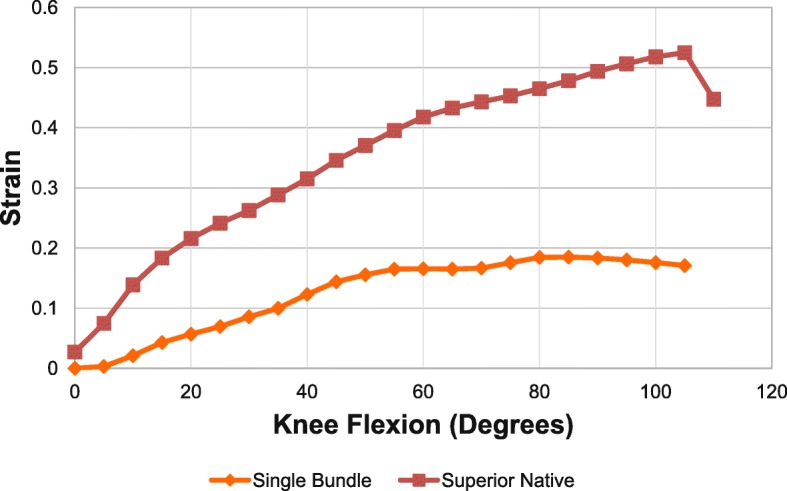
Strain pattern of intact and single-bundle reconstructed MPFL as knee flexed from 0 to 105 degrees. Red – superior intact, Orange – single bundle

Figure 7 shows the comparison of strain for the superior band of the native MPFL and single bundle MPFL reconstructed specimens. Only the superior strain was compared as there was no statistical difference between the superior and inferior aspects of the native MPFL.

### Intact MPFL VS single bundle reconstructed MPFL

The strain in the single bundle was significantly lower than the superior native MPFL. This was statistically significant at flexion angles of 45 degrees and above as shown in Table 2.

**Table 2:** Native superior vs Single bundle strain measurements (*n* = 6)

Flexion °	Native Superior	Single Bundle	*P*-value
30	0.26 ± 0.17	0.09 ± 0.05	0.053
45	0.35 ± 0.15	0.14 ± 0.03	0.021
60	0.42 ± 0.14	0.17 ± 0.04	0.005
90	0.49 ± 0.10	0.18 ± 0.04	< 0.001
105	0.52 ± 0.12	0.17 ± 0.06	< 0.001

### Superior bundle of double bundle VS single bundle reconstructed MPFL

**Fig. 8:**
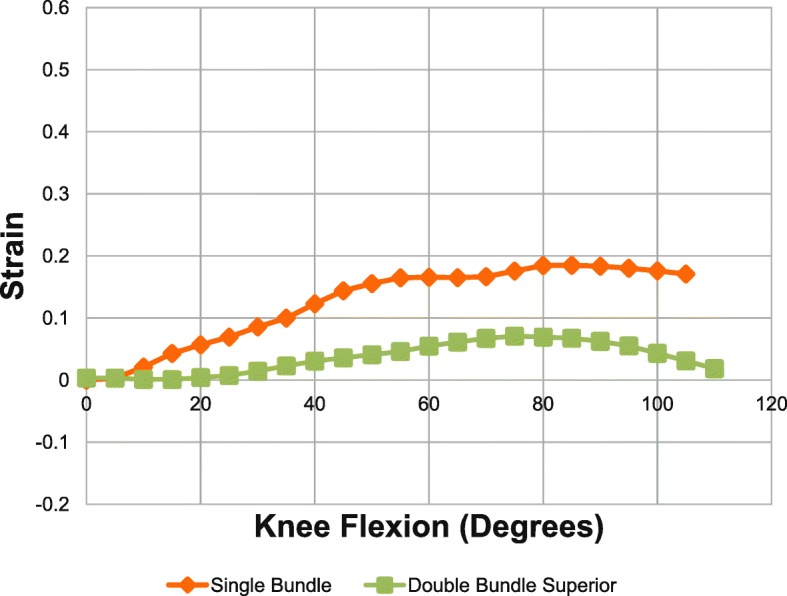
Strain pattern of single-bundle and superior band of double-bundle MPFL as knee flexed from 0 to 105 degrees. Green – superior reconstructed, Orange – single bundle reconstructed

Figure 8 shows the comparison of the graft of the single bundle reconstructed specimen and the superior bundle of the double bundle MPFL reconstructed specimen. Since the inferior bundle of the double-bundle group showed loss of tension (negative strain), it was not used in the comparison. The single bundle reconstructed specimen showed a higher strain with statistical significance at flexion angles less than 90 degrees as shown in Table 3.

**Table 3:** Strain in superior band of double bundle versus single bundle MPFL reconstruction (*n* = 6)

Flexion °	Superior band of double bundle	Single Bundle	*P*-value
30	0.01 ± 0.03	0.09 ± 0.05	0.022
45	0.04 ± 0.02	0.14 ± 0.03	< 0.001
60	0.05 ± 0.02	0.17 ± 0.04	< 0.001
90	0.49 ± 0.10	0.18 ± 0.04	< 0.001
105	0.03 ± 0.13	0.17 ± 0.06	0.066

## Discussion

Our literature review showed only one study that evaluated the strain within the MPFL. McCulloch et al. compared the strain behaviour of the native and reconstructed MPFL using a visible-light stereophotogrammetry (VLS) system (McCulloch et al. 2014). The VLS system used markers attached to the soft tissues to obtain stereoscopic images to measure the strain. In this study, a customized jig was able to simulate joint weight-bearing. Their study was not a dynamic study as they studied the strain pattern at 10 different flexion angles. They found that the MPFL strain increased linearly with knee flexion with a maximum strain at 120 degrees. Although they did double-bundle MPFL reconstructions, they reported the averaged reading of the two bundles instead of measuring the strain on each of the bundles. They observed that the average strain of the reconstructed MPFL was significantly less than the native MPFL. Similar to our study, they found an abrupt increase in strain between 25 and 30 degrees of knee flexion. This would correspond to the patella engaging in the trochlea groove. Although their study was the first to investigate strain within the MPFL and compare it to the reconstructed MPFL, they did not compare the regional variations of strain.

As compared to McCulloch et al., our experimental set up was able to successfully capture the strain behaviour of the native intact MPFL and compare it to the strain in each of the bundles of the double-bundle reconstruction and the single-bundle reconstruction as the knee flexes and extends dynamically. Our study did not simulate weight-bearing.

In the native MPFL the maximum increase in strain is observed around 30 degrees of flexion which corresponds with the engagement of the patella within the trochlear groove. This is observed over both the superior and inferior aspect of the intact MPFL. This however is not reproduced in the reconstructed group. The superior band showed a linear increase in strain until about 90 degrees of flexion before decreasing. This observation suggests that engaging in the trochlear groove did not affect the strain patterns in the reconstructed MPFL likely due to the increased stiffness of the grafts.

Our study also shows that the superior band of the reconstructed MPFL was up to five times stiffer at maximum strain compared to the native MPFL. Feller et al. demonstrated that the hamstring graft was less elastic compared to the native MPFL (Feller et al. 2007). This would explain the observed difference in strain behaviour between the tissues.

An interesting observation in all six specimens with double-bundle reconstruction was that the inferior graft showed negative strain suggesting that there was loss in tension as the knee was flexed. The superior graft underwent tensile deformation and recorded a positive strain. Based on the above reproducible observation we gather that the inferior band may be redundant. We postulate that the increased stiffness of the superior bundle resulted in the loss of tension of the inferior bundle. We also postulate that the increased stiffness may result in the rotation of the patella as the knee is flexed resulting in a loss of tension in the inferior bundle of the reconstructed group. Further studies will need to analyse the rotation of the patella as it engages with the trochlea groove.

Other studies have been conducted which compared single-bundle and double bundle MPFL reconstruction. Wang et al. studied the force required to dislocate the patella at various flexion angles after single and double-bundle reconstruction. They concluded that only at 15 degrees of flexion was the force statistically significantly higher in the double-bundle group (Wang et al. 2017). Wang et al. showed better clinical outcomes (outcome scores and lower re-dislocation rates) in double-bundle reconstruction versus single bundle reconstruction (Wang et al. 2013). No studies have compared the strain behaviour within single and double-bundle MPFL reconstruction to native MPFL. Our study shows that single-bundle MPFL reconstruction exhibits a better strain profile compared to double-bundle reconstruction. A systematic review by Kang et al. (2019) showed that single-bundle reconstruction demonstrated similar outcomes with regards to re-dislocation rates, functional scores and complications compared to double-bundle reconstruction. The single-bundle group had a greater risk of post-operative apprehension while the double-bundle group had increased post-operative stiffness (Kang et al. 2019).

The strain variation at progressive angles of knee flexion is dissimilar between the native and reconstructed MPFL. Based on the findings, the reconstructed MPFL exhibits non-physiological biomechanics.

This study exhibited various strengths. This is the first study to compare the strain behaviour of native, single-bundle and double-bundle MPFL reconstructions. The strain within the superior and inferior reconstructed bundles were assessed individually. By using dynamic flexion, we were able to compare a large number of flexion angles from full extension to deep flexion. The DVRTs ensured precise measurements of strain at various flexion angles were easily reproducible. Since the sectioning and the reconstruction of the MPFL were performed by a single surgeon using a standardized technique, the risks of intra-observer and technical error were minimized.

The reconstruction of the MPFL was performed using the semitendinosus rather than the gracilis tendon. Noyes et al. studied the biomechanical properties of semitendinosus and gracilis tendons. The gracilis tendon had a lower stiffness (482.8 KN/m) compared to the semitendinosus tendon (559.5 KN/m), however the surface strain and grip-to-grip strain between these tissues showed no statistical significant difference (Noyes et al. 1984). Despite the gracilis tendon having a lower stiffness, we used the semitendinosus due to the longer graft lengths and consistency in width. A systemic review performed by McNeilan et al. showed semitendinosus autograft had slightly lower recurrent instability (0.6%) compared to gracilis (2.0%) and higher improvement in Kujala Scores (McNeilan et al. 2018).

Our study had some limitations as listed below:-

1. The mean age of the cadaveric knees was 55 years old. The strain measurements may not be extrapolated to the younger patient population that commonly have recurrent patellar dislocation (Fithian et al. 2004).
2. The tension with which the grafts were tightened may not be the same in all specimens as a tension gauge was not used. This however will not contribute to the difference between the superior and inferior bands as they were both tensioned at the same time using the Bio-Interference screws with the knee at 30 degrees flexion. The grafts should have been tensioned at a fixed load of 2 N as described by Melegari et al. (Noyes et al. 1984). In clinical practice the grafts are tensioned by keeping the lateral patellar facet flush against the lateral femoral condyle. The same technique was employed.
3. The study uses fresh-frozen cadavers which may not exhibit the same material properties as in-vivo tissues. Other in-vivo effects cannot be taken into consideration such as graft remodelling and healing.
4. In the single-bundle reconstructed knee not all 6 knee specimens were able to achieve 120 degrees of flexion. Due to this reason the statistical analysis was performed for flexion angle 0 to 105 degrees.

## Conclusion

MPFL reconstruction does not replicate the strain patterns of the native MPFL. The reconstructed grafts show significantly lower strains and thus are far stiffer.

Double-bundle reconstructions replicate the native anatomy in terms of recreating the fan-shape, but exhibit strain patterns inferior to single bundle reconstructions. The strain difference between the superior and inferior grafts of the double-bundle reconstruction is a concern.

There is a need to explore other graft choices, surgical techniques and fixation methods that can lead to better restoration of native MPFL biomechanics.
